# Annotationum Academicarum Fasciculi I. et II

**Published:** 1782

**Authors:** 


III. Georgii Prochaſka, M. D. Anatom. et de Morb. Oculor. Profeſſoris, &c. *Annotationum Academicarum Faſciculi* I. *et* II. 4to. Prague, 1780 and 1781.

THE firſt of theſe collections is divided into two ſections. In the firſt ſection the author preſents us with ſeveral ingenious obſervations relative to the decreaſe of the teeth in the human body.

He finds that the inciſores, particularly the two middlemoſt, are the firſt that experience a diminution, becauſe they are naturally ſharper than the other two, and this diminution, we are told, gradually becomes ſo conſiderable, that in elderly people two thirds of theſe teeth are worn away. The inner cavity of the teeth is obliterated in proportion as this diminution takes place, being gradually filled up by the bony ſubſtance.

Our author has obſerved, that at the age of twenty years the canine teeth in the generality of

ſubjects are ſo much worn down, that the nucleus of their bony ſubſtance is diſtinguiſhable. The molares, we are told, reſiſt a longer time.

Theſe remarks lead him to aſk, whether the teeth may not ſerve to determine the age of the human ſpecies as well as of horſes? He is for the affirmative, but allows that a multitude of circumſtances may render our knowledge on this head very doubtful.

He next inquires into the uſe of the enamel, and this he thinks is not yet ſatisfactorily aſcertained. He rejects the opinion, that the teeth are conſtantly growing.

The ſecond faſciculus contains five ſections. In the firſt we have an account of a biliary calculus voided by ſtool. The patient, a woman forty years old, after having been long ſubject to cardialgia, which was generally relieved by vomiting, one day felt as if ſomething was detached from the pit of her ſtomach. From that inſtant the cardialgia left her, and inſtead of it ſhe complained of pain in the left hypochondrium. She continued to be ſubject to this pain, to jaundice, and a variety of other troubleſome ſymptoms, for the ſpace of about three years; at the end of which time the pain ſuddenly left her, her, and ſoon after ſhe voided this gallſtone, and recovered her health perfectly.

Our author ſuſpects, that the greater or leſs facility with which theſe biliary concretions diſſolve by heat, depends on the quantity of fixed air they contain.

In the ſecond ſection we have an account of two caſes, both of which terminated fatally. The ſubject of one of theſe was a woman, about fifty years old, who laboured under all the ſymptoms of a ſchirrous uterus. After death he found the pelvis filled with ſchirrous tumours, which puſhed the urinary bladder towards the pubis, in the ſame manner as the uterus does during pregnancy. The ileon was found adhering to the fundus uteri. The whole of the right Fallopian tube was ſchirrous; the left was in a healthy ſtate, but the inner ſurface of the uterus was ulcerated. The uterus was conſiderably diſtended, and the bladder at its poſterior ſurface ſchirrous.

The ſubject of the ſecond caſe was a woman, thirty years old, who for ſome time had been afflicted with ſcrophulous tumours of the neck, obſtinate eruptions in the face, head-ach, profuſe fluor albus, and frequent uterine hæmorrhage.

On

On diffection the iliac lymphatic glands were found enlarged to the fize of a fift.

In the third fection the author fpeaks of a tumour in the right hypochondrium, which occurred in a woman twenty-five years of age, and was at firft confidered as a difeafe of the right ovarium. She had laboured under it about fifteen weeks when he firft faw it, and its bulk was then equal to that of two fifts. Recourfe was had to the frequent repetition of mild purges, and externally to the ufe of unctuous applications, and in about three months the tumour fubfided. From its mode of termination, our author fuppofes it to have been occafioned by a collection of fæces in the cœcum.

The fourth fection contains defcriptions of four monfters; and in the fifth and laft the author offers fome obfervations on the different fyftems of generation.

